# The relationship between weight locus of control, eating behaviors, and anthropometric measurements in menopausal women aged 45–55: a cross-sectional study

**DOI:** 10.1186/s12905-026-04426-x

**Published:** 2026-04-01

**Authors:** Ceren Şarahman-Kahraman, Cansu Memi̇ç-İnan, Sadiye Yeşi̇lkir, Meltem Soylu

**Affiliations:** 1 Department of Nutrition and Dietetics, University of Alanya Alaaddin Keykubat, Kestel District, University Street, No:80, Alanya, 07425 Antalya Turkey; 2 Department of Nutrition and Dietetics, University of Hitit, Gazi Street, No:86, Çorum, 019200 Turkey

**Keywords:** Anthropometry, Eating behavior, Emotional eating, Menapouse, Weight locus

## Abstract

**Background:**

Weight locus of control (WLOC), which reflects individuals’ beliefs about weight control, may be an important determinant of eating behaviors and body composition. This study aims to examine the relationship between WLOC, eating behaviors, and anthropometric measurements in menopausal women.

**Methods:**

The sample for this cross-sectional correlational study consists of 300 menopausal women aged 45–55 living in Alanya. Study data were collected through face-to-face interviews using a questionnaire. The questionnaire included general information and anthropometric measurements, the Turkish version of the Multidimensional Weight Locus of Control Scale, and the Three-Factor Eating Questionnaire.

**Results:**

Of the participants, mean body mass index 25.8 ± 6.2 kg/m², and 67.7% had a body fat percentage (BF%) ≥ 25%. In overweight and obese, internal control scores were lower, while uncontrolled and emotional eating scores were higher (*p* < 0.05). According to the logistic regression analysis, as internal control locus increased, waist-to-hip ratio (OR = 0.888) and BF% (OR = 0.923) decreased, whereas an increase in chance locus (OR = 1.080) and uncontrolled eating (OR = 1.117) was associated with higher body mass index (BMI) (*p* < 0.05).

**Conclusion:**

It was determined that WLOC is associated with eating behaviors and anthropometric measurements in menopausal women. The results highlight the importance of identifying WLOC in menopausal women and tailoring weight management programs accordingly.

## Introduction

Menopause is an endocrinological transition process characterised by the permanent cessation of the menstrual cycle due to the reduction of ovarian follicles in women, signifying post-reproductive life, and is generally observed between the ages of 45 and 55 [1, 2]. During this process, the incidence of various chronic diseases such as cardiovascular disease, psychological stress, and increased central obesity increases due to the decline in oestrogen levels [3]. During menopause, insufficient oestrogen levels affect the central nervous system, altering food intake and basal metabolic rate. With the disappearance of oestrogen’s effect, basal metabolism slows down and energy intake increases as a result of the decrease in oestrogen’s appetite-suppressing effect on alpha receptors in the central nervous system [4]. Women in menopause experience more intense cravings and hunger signals for foods that lead to weight gain (WG) [5]. In addition, changes in hormone balance lead to a decrease in basal metabolic rate, increasing total fat mass and visceral fat while decreasing muscle mass [6]. In short, during the menopause, body weight (BW) increases, and changes occur in body composition.

It is reported that 70% of women gain weight during the menopausal transition and their body mass index (BMI) increases, continuing into the postmenopausal period [7]. WG associated with both chronological ageing and menopause is observed in middle-aged women, and it is stated that WG is a serious cause for concern for women during this period [6]. A meta-analysis examining changes in fat mass in menopausal women reported that attention should be paid to postmenopausal central obesity and that total fat mass should be monitored throughout life [8]. Therefore, women going through the menopause should pay attention to eating habits and weight control that may affect their BW.

Appetite regulation is affected by changes in oestrogen hormone activity in women and eating habits may differ compared to before menopause. It is noted that WG during menopause, changes in body composition, and depressive mood may lead to abnormal eating behaviors such as overeating or restrictive eating [9]. Furthermore, the frequent observation of stress and mood swings in middle-aged women may result in stress-related emotional eating (EE) [10, 11]. Therefore, examining eating behavior, which is a modifiable behavior, is crucial for achieving weight control during menopause. When all these factors are considered together, it is thought that the locus on weight control is an issue that should not be overlooked.

A person’s belief in their ability to control certain events in their life is referred to as locus of control and is an important concept in weight management [12]. If an individual believes that they control certain events based on their behavior or consistent characteristics, this is referred to as an internal locus of control, and internal locus of control is a highly influential variable in weight control [13]. Furthermore, as it has been noted that individuals with an internal control locus may consciously exhibit restricted eating behavior, it is considered necessary to evaluate the cognitive aspects of eating behavior during menopause. When combined with environmental food cues, the emotional changes experienced during this process can lead individuals to consume food, which may be related to their weight locus of control (WLOC) [14]. Individuals with an external locus of control, on the other hand, attempt to manage their BW with professional support and believe that weight control is shaped by chance, healthcare professionals, or other peoples. When constructive support is received, it is reported that external locus of control has positive effects on weight management and health [13].

With increased life expectancy, women spend approximately one-third of their lives in the menopausal period [15]. It is known that menopause affects women physically, psychologically, and metabolically, and that these changes, when combined with ageing, can result in WG. For this reason, women undergoing menopause represent a special population that requires particular attention regarding weight management. Although behavioral and cognitive factors affecting weight management during menopause have been studied [16], research examining the relationship between a WLOC and anthropometric measurements is limited and has primarily targeted the general adult population [14, 17, 18]. Furthermore, although previous studies have examined eating behavior and diet quality in relation to anthropometric measurements in menopausal women [19, 20], evidence regarding the relationship between a WLOC and anthropometric measurements in menopausal women aged 45–55 remains limited. Evaluating WLOC may provide valuable insights for the development of targeted interventions in menopausal weight management. Accordingly, this study aimed to determine the WLOC of menopausal women and to investigate its relationship with their eating behavior and anthropometric measurements.

## Method

### Design and participants

The sample for this study consists of women aged between 45 and 55 who are going through menopause and who receive support from the Alanya Municipality’s first-degree healthcare service provider, Accessible Park and Life Center, and the Women’s Policy and Family Support Center, located in the Alanya district of Antalya province. Sample size calculation was performed using G*Power analysis, referencing relevant literature [21–23]. Assuming a medium effect size (Cohen’s d = 0.30), a power of 90%, and a Type I error rate of 5%, the minimum required sample size was calculated as 230 women. The research data was collected between November 2024 and March 2025, and 300 women in menopause participated in the study.

### Inclusion criteria

- Being 45–55 years of age.
- Having undergone natural menopause (≥ 12 months of amenorrhea).
- Living in Alanya district.
- Benefit from the Accessible Park and Life Center, Women’s Policy and Family Support Center.
- Being literate and voluntarily agreeing to participate in the work.

### Exclusion criteria

- Having undergone menopause due to surgical or medical causes.
- Being in the perimenopausal period.
- Receiving medical treatment that may affect BW.
- Having an eating disorder (anorexia, bulimia nervosa, etc.)

The research data was collected using a face-to-face interview method with a questionnaire prepared by the researcher. Prior to the collection of study data, ethical committee approval was obtained from the Non-Interventional Clinical Research Ethics Committee of Alanya Alaaddin Keykubat University, dated 12 November 2024, and bearing decision number 2024/09. The study was conducted in accordance with the Helsinki Declaration and informed consent was obtained from all women.

### Measures

#### General information

This section contains information on women’s age, income level, education level, chronic illnesses, exercise and dietary habits. Individuals’ regular exercise status were determined using a multiple-choice question asking whether they engage in at least 150 min of moderate-intensity activity (brisk walking, swimming, etc.) per week.

#### Multidimensional Weight Locus of Control Scale (T-MWLCS)

The T-MWLCS, developed by Cebolla et al. [18] and validated in Turkish by Kahraman & Ayhan [14], was used to measure individuals’ beliefs about how they control their BW. The scale consists of 18 items and 4 subfactors. The internal subfactor consists of items 1, 6, 8, 12, and 17; the chance subfactor consists of items 2, 4, 9, 11, 15, and 16; the doctors subfactor consists of items 3, 5, 13, and 14; and the other people subfactor consists of items 7, 10, and 18. The 6-point Likert-type scale is scored as follows: 0 = strongly disagree, 1 = mostly disagree, 2 = slightly disagree, 3 = slightly agree, 4 = mostly agree, and 5 = strongly agree. The scale yields a minimum score of 0 and a maximum score of 90. The Cronbach’s alpha values for the sub-factors of the T-MWLCS scale (internal, chance, doctors, other people, respectively) are 0.72, 0.86, 0.66 and 0.69. Although there are no reverse-coded items on the scale, it is indicated that whichever sub-factor has a high score, the person’s locus of control regarding their BW is in that direction.

#### Three-Factor Eating Questionnaire (TFEQ-R21)

To measure the behavioral and cognitive components of eating, the 51-item scale developed by Stunkard & Messick [24] was modified due to limited applicability, and Karlsson et al. [25] reduced the number of items to create the final 21-item form. The Turkish adaptation of the scale was conducted by Karakuş et al. [26] and is scored using a 4-point Likert scale (1 = definitely false, 2 = mostly false, 3 = mostly true, and 4 = definitely true). The scale is composed of three subfactors: cognitive restraint (CR), emotional eating (EE), and uncontrolled eating (UE). The UE subfactor consists of items 3, 6, 8, 9, 12, 13, 15, 19, and 20; the CR subfactor consists of items 1, 5, 11, 17, 18, and 21; and the EE subfactor consists of items 2, 4, 7, 10, 14, and 16. The UE subfactor can yield a minimum of 9 and a maximum of 36 points, while the CR and EE subfactors can yield a minimum of 6 and a maximum of 24 points. The Cronbach’s alpha coefficient for the TFEQ-R21 scale was determined to be 0.801 for the CR sub-factor, 0.870 for EE, and 0.787 for UE. In the scale, higher scores on a given sub-factor indicate a more dominant eating behavior associated with that factor.

#### Anthropometric measurements

The researchers took measurements of height (cm), BW (kg), waist circumference (cm) and hip circumference (cm) of women. Height, waist, and hip circumferences were measured using a non-stretch tape measure on the Frankfurt plane, and BW was measured using a Tanita BC 401 scale [27]. BMI was calculated using BW and height measurements and was assessed according to WHO criteria: 18.50–24.99 kg/m² normal, 25.0–29.99 kg/m² overweight, ≥ 30.00 kg/m² obese. The waist-to-hip ratio (WHR) was calculated by dividing the waist circumference by the hip circumference, and a value of ≥ 0.85 in women was classified as high risk [28].

#### Body composition

The body composition of the women was determined using bioelectrical impedance analysis with a Tanita BC 401. To ensure accurate measurements, participants were required not to have consumed any food or drink for at least four hours prior, to have refrained from exercise and alcohol consumption the day before, and not to be wearing any metal accessories [29]. BF% for women were evaluated according to the recommendation of the Turkish Nutrition Guide-2022 [30].

### Data analysis

The data obtained from the study were analyzed using version 26 of the Statistical Package for the Social Sciences (SPSS) software. The conformity of the variables in the dataset to a normal distribution was determined using hypothesis tests. Descriptive data were presented using frequency and percentage tables. For the evaluation of categorical variables, Pearson’s chi-square test was used when the expected frequency was greater than 0.20, and Fisher’s exact test was applied when the expected frequency was less than 0.20. Variables with a normal distribution were presented as mean and standard deviation, whereas those not normally distributed were presented as median, minimum, and maximum values. The significance of the difference between the two groups exhibiting a normal distribution was determined using the independent samples t-test. Binary logistic regression analysis was performed to identify potential predictors of high BMI (≥ 25 kg/m^2^), WHR (≥ 0.85), and high BF% (> 25%). Model development was guided by previous literature and theoretical considerations. Variables known or suspected to be associated with both the exposure and outcome were identified as potential confounders and included in the models. Therefore, adjustments were made for age, marital status, education level, presence of chronic disease, regular exercise, and history of weight-loss dieting. The findings were evaluated at the 0.05 level of significance.

## Results

The general information about the participants is presented in Table 1. Most participants (60%) were aged 50–55 years (mean 50.2 ± 3.1), married (76.3%), and primary school graduates (61.3%). Over half of the participants reported middle income (57%) and chronic disease (54%), with diabetes being the most common (45.7%). Most participants had previously followed a weight-loss diet (78.7%), while only 22.3% exercised regularly. The mean BMI was 25.8 ± 6.2 kg/m^2^, and 53.0% were overweight or obese (BMI ≥ 25 kg/m^2^). A high WHR (≥ 0.85) was identified in 13.0% of the participants, while 67.7% were found to have a high BF% (> 25%). Mean T-MWLCS scores were 17.5 ± 5.6 (internal), 10.3 ± 7.5 (chance), 9.7 ± 4.6 (doctors), and 7.4 ± 4.1 (other people). Mean TFEQ-R21 scores were 17.5 ± 6.0 for UE, 14.5 ± 3.8 for CR, and 12.7 ± 5.1 for EE.

**Table 1:** General information about participants

Characteristic	Numbers (%)
	Mean ± SD
Age (years)	50.2 ± 3.1
45–49	120 (40.0)
50–55	180 (60.0)
Marital status, *n* (%)	
Single	71 (23.7)
Married	229 (76.3)
Education level, *n* (%)	
Primary education	184 (61.3)
High school	54 (18.0)
University	62 (20.7)
Income (monthly)	
Low	104 (34.7)
Middle	171 (57.0)
High	25 (8.3)
Chronic Diseases	
Yes	162 (54.0)
No	138 (46.0)
Distribution of Chronic Diseases*	
Diabetes	69 (45.7)
Hypertension	30 (19.9)
Cardiovascular diseases	25 (16.6)
Respiratory system diseases	14 (9.3)
Digestive system disorders	18 (11.9)
Musculoskeletal disorders	27 (17.9)
Previous history of weight loss diets	
Yes	236 (78.7)
No	64 (21.3)
Regular Exercise Status	
Yes	67 (22.3)
No	233 (77.7)
BMI (kg/m^2^)	25.8 **±** 6.2
< 25 kg/m^2^	141 (47.0)
≥ 25 kg/m^2^	159 (53.0)
WHR	
Normal	261 (87.0)
High (≥ 0,85 cm)	39 (13.0)
Body fat (%)	
Normal	97 (32.3)
High (>%25)	203 (67.7)
T-MWLCS score	
Internal	17.5 ± 5.6
Chance	10.3 ± 7.5
Doctors	9.7 ± 4.6
Other people	7.4 ± 4.1
TFEQ-R21 score	
UE	17.5 ± 6.0
CR	14.5 ± 3.8
EE	12.7 ± 5.1

Note: Age categorized by median

*BMI* Body mass index, *T-MWLCS* Multidimensional weight locus of control scale, *TFEQ-R21* Three-Factor eating questionnaire, *WHR* Waist to hip ratio, *UE* Uncontrolled eating, *CR* Cognitive restraint, *EE* Emotional eating

* Multiple answers have been selected

Table 2 provides general information about the participants according to their BMI classification. Low income (*p* = 0.022) and prior weight-loss dieting (*p* < 0.001) were more common among overweight/obese participants than those of normal weight. The frequency of regular exercise is significantly higher in the normal BMI (*p* < 0.001). Overweight/obese participants had lower internal control scores (*p* < 0.001) but higher chance (*p* < 0.001), doctors (*p* = 0.003), and other people scores (*p* < 0.001). Overweight/obese participants scored higher on uncontrolled (*p* < 0.001) and EE scores (*p* < 0.001), while no difference was observed between groups in terms of CR (*p* > 0.05).

**Table 2:** General information about participants according to BMI classification

	BMI Classification		*p*
	< 25 kg/m^2^	≥ 25 kg/m^2^	
Age (years)	50.0 ± 3.0	50.4 ± 3.2	0.340
45–49	61 (43.3)	59 (37.1)	0.277
50–55	80 (56.7)	100 (62.9)	
Marital status			
Married	105 (74.5)	124 (78.0)	0.474
Single	36 (25.5)	35 (22.0)	
Education status			
Primary education	78 (55.3)	106 (66.7)	0.116
High school	28 (19.9)	26 (16.4)	
University	35 (24.8)	27 (17.0)	
Income (monthly)			
Low	43 (30.5)	61 (38.4)	**0.022**
Middle	80 (56.7)	91 (57.2)	
High	18 (12.8)	7 (4.4)	
Chronic Disease			
Yes	77 (54.6)	85 (53.5)	0.842
No	64 (45.4)	74 (46.5)	
Previous history of weight loss diets			
Yes	88 (62.4)	148 (93.1)	**< 0.001**
No	53 (37.6)	11 (6.9)	
Regular Exercise Status			
Yes	46 (32.6)	21 (13.2)	**< 0.001**
No	95 (67.4)	138 (86.8)	
T-MWLCS			
Internal	19.5 ± 4.4	15.8 ± 5.9	**< 0.001**
Chance	7.1 ± 6.4	13.3 ± 7.2	**< 0.001**
Doctors	8.8 ± 4.5	10.4 ± 4.6	**0.003**
Other people	5.8 ± 4.3	8.8 ± 3.3	**< 0.001**
TFEQ-R21			
UE	14.5 ± 3.8	20.1 ± 6.4	**< 0.001**
CR	14.9 ± 3.9	14.2 ± 3.7	0.138
EE	10.3 ± 3.9	14.9 ± 5.1	**< 0.001**

Independent sample t-test, Pearson’s chi-square test. Statistically significant data is shown in bold

*BMI* Body mass index, *T-MWLCS* Multidimensional weight locus of control scale, *TFEQ-R21* Three-Factor Eating questionnaire, *UE* Uncontrolled eating, *CR* Cognitive restraint, *EE* Emotional eating

Table 3 shows the correlations between the study variables. BMI was found to be positively associated with BF%, WHR, chance, other people, UE and EE (respectively *r* = 0.717, *r* = 0.304, *r* = 0.434, *r* = 0.401, *r* = 0.480, *r* = 0.435; all p values *p* < 0.01), and negatively associated with internal control factor (*r*=-0.428, *p* < 0.01). BF% was found to be negatively associated with internal control (*r*=-0.360, *p* < 0.01), and positively associated with chance, other people, UE, and EE (respectively *r* = 0.234, *r* = 0.306, *r* = 0.307, *r* = 0.288; all p values *p* < 0.01). WHR is negatively correlated with internal control (*r*= -0.231, *p* < 0.01) and positively correlated with UE (*r* = 0.115, *p* < 0.05).

**Table 3:** Relationships between the variables of the study

	BMI (k/m^2^)	Body fat (%)	WHR	Internal	Chance	Doctors	Other people	UE	CR	EE
BMI	1	0.717**	0.304**	-0.428**	0.434**	0.097	0.401**	0.480**	-0.086	0.435**
Body fat (%)		1	0.396**	-0.360**	0.234**	0.079	0.306**	0.307**	0.03	0.288**
WHR			1	-0.231**	0.049	-0.104	-0.008	0.115*	-0.016	0.057
Internal				1	-0.501**	0.335**	-0.261**	-0.376**	0.174**	-0.281**
Chance					1	-0.005	0.430**	0.426**	-0.189**	0.409**
Doctors						1	0.336**	0.175**	0.251**	0.209**
Other people							1	0.359**	-0.128*	0.351**
UE								1	-0.097	0.670**
CR									1	-0.095
EE										1

*BMI* Body mass index, *T-MWLCS* Multidimensional weight locus of control scale, *TFEQ-R21* Three-Factor eating questionnaire, *WHR* Waist to hip ratio, *UE* Uncontrolled eating, *CR* Cognitive restraint, *EE* Emotional eating

Pearson correlation test, *p < 0.05, **p < 0.001

Internal control correlated with chance (*r*=-0.501), doctors (*r* = 0.335), other people factor (*r*=-0.261), UE (*r*=-0.376), CR (*r* = 0.174), and EE (*r*=-0.281) (all *p* < 0.01). The chance factor correlated with other people (*r* = 0.430), UE (*r* = 0.426), CR (*r*=-0.189), and EE (*r* = 0,409), whereas the doctor factor correlated with other people (*r* = 0.336), UE (*r* = 0.175), CR (*r* = 0.251), and EE (*r* = 0.209 (all *p* < 0.01).

The results of the binary logistic regression analysis evaluated potential predictors of high BMI (≥ 25 kg/m²), high WHR (≥ 0.85), and high BF% (> 25%) (Table 4). For high BMI, chance (OR = 1.080, 95% CI:1.019–1.146; *p* = 0.010) and UE (OR = 1.117, 95% CI:1.041–1.199; *p* = 0.002) were identified as significant predictors. For high WHR, only internal emerged as a significant predictor, with higher internal scores associated with a lower risk of elevated WHR (OR = 0.888, 95% CI:0.817–0.966; *p* = 0.006). Similarly, for high BF%, internal was a significant negative predictor, indicating that higher internal scores reduced the likelihood of having an elevated BF% (OR = 0.923, 95% CI:0.858–0.992; *p* = 0.030).

**Table 4:** Predictors of High BMI, WHR, and Body Fat (%) – Binary Logistic Regression

Variable	Odds Ratio	95% CI for odds ratio		*p*
		Lower	Upper	
High BMI (≥ 25 kg/m²)				
Internal	0.935	0.869	1.005	0.068
Chance	1.080	1.019	1.146	**0.010**
Doctors	1.043	0.952	1.142	0.369
Other people	1.000	0.906	1.103	0.995
UE	1.117	1.041	1.199	**0.002**
CR	0.957	0.879	1.041	0.304
EE	1.063	0.987	1.145	0.104
High WHR (≥ 0,85 cm)				
Internal	0.888	0.817	0.966	**0.006**
Chance	0.960	0.899	1.025	0.224
Doctors	0.803	0.910	1.129	0.803
Other people	1.028	0.908	1.164	0.661
UE	0.990	0.913	1.074	0.811
CR	1.026	0.930	1.132	0.609
EE	1.014	0.921	1.116	0.783
High body fat (%) (> %25)				
Internal	0.923	0.858	0.992	**0.030**
Chance	0.988	0.937	1.043	0.666
Doctors	1.038	0.955	1.127	0.380
Other people	1.075	0.985	1.173	0.103
UE	1.042	0.972	1.116	0.247
CR	1.065	0.983	1.153	0.122
EE	1.076	0.996	1.163	0.065

Adjusted for: age, marital status, educational status, chronic illness, exercise, previous weight loss diet

*BMI* Body mass index, *WHR* Waist to hip ratio, *UE* Uncontrolled eating, *CR* Cognitive restraint, *EE* Emotional eating, *CI* Confidence interval

Binary Logistic Regression, *p* < 0.05

## Discussion

This study was conducted to determine the relationship between WLOC, eating behaviour and anthropometric measurements in menopausal women. The study findings demonstrated that increases in BMI, WHR, and BF% among women were associated with lower levels of internal WLOC and higher levels of external WLOC, including chance and other people. In addition, it was determined that women with high BMI and BF% exhibited higher levels of UE and EE, while women with high WHR showed higher levels of UE. Our study found that internal WLOC was a significant negative predictor of both BF % and high WHR. Chance WLOC and UE were identified as significant predictors of high BMI. Overall, these findings suggest that different types of WLOC may be related to anthropometric measurements, potentially through eating behaviors.

It is known that WG and changes in body composition occur in middle-aged women during the menopausal transition [8, 31]. Therefore, with advancing age, menopause places women in a more vulnerable population in terms of obesity and adverse health outcomes [32]. In our study, BMI was found to have a strong positive correlation with BF% and a moderate positive correlation with WHR. This finding is consistent with the literature indicating increases in BMI and total BF% during menopause [33]. The moderate relationship observed between BMI and WHR in our study may support the literature [34] suggesting that fat gain during menopause is not only associated with increased BW but also with the accumulation of fat in the abdominal region. Therefore, during menopause, it is essential to assess not only BMI but also body composition [35].

Since the menopausal period is a unique phase in which women experience WG and abdominal fat accumulation, and concerns about WG increase, assessing WLOC may be important for BW management [6]. WLOC assesses a person’s belief in their ability to control their BW [18], and the positive effect of an internal WLOC on BW management has been reported [12, 13]. In our study, women with a BMI ≥ 25 kg/m^2^ were found to have lower internal WLOC, higher WLOC—such as chance, doctors, and other people—and higher frequency of diet adherence. Although there is limited information on overweight individuals and WLOC, it has been reported that obese individuals exhibit a higher external WLOC compared to those who are not obese [36]. The value of measuring WLOC in obesity research has not yet been fully understood, and to our knowledge, no studies have evaluated WLOC specifically during menopause. Studies have generally associated WLOC with weight loss programs, and a positive effect of internal WLOC on maintaining weight loss has been observed [13, 36, 37]. This finding may suggest that lower internal WLOC is associated with difficulties in sustaining dietary behaviors among women with BMI ≥ 25 kg/m^2^.

In this study, a negative relationship was observed between BMI and internal WLOC, while positive relationships were found between BMI and the factors of chance and other individuals. This indicates that individuals with high BMI have insufficient belief in their own influence over their weight (low internal WLOC), while they have a stronger belief that their weight is affected by chance or other people (external WLOC). Our finding supports the literature [38, 39] indicating that individuals with high BMI may have a higher external WLOC. During menopause, it is believed that priority should be given to web-based or public health campaigns aimed at promoting internal WLOC, where individuals’ self-efficacy in weight management is emphasized, to prevent excessive BW.

In women, eating behaviors vary during periods of hormonal, physical, and psychosocial changes, such as adolescence, pregnancy, and breastfeeding [40]. In fact, during menopause, hormonal changes may lead to increased appetite and alterations in eating behaviors [41]. In our study, a strong relationship was observed between BMI and both UE and EE. Additionally, a significant association was found between WHR and UE. This finding is consistent with the literature linking EE and UE to obesity in women [42]. In a study examining obese adults with a BMI ≥ 35 kg/m^2^, similar to our findings, it was reported that 50% of the participants had high EE scores, and 25% had high UE scores [43]. In another study evaluating the relationship between UE and EE with BMI in women, it was reported that UE and EE may contribute to higher BMI [44]. These findings indicate that EE and UE behaviors are associated with WG indicators during menopause. Supporting emotion regulation skills during menopause is thought to help prevent both adverse eating behaviors and increases in BMI.

The negative relationship between internal WLOC and UE and EE, along with its positive relationship with CR observed in this study, may suggest that individuals who perceive themselves as having control over their BW tend to eat in a planned and controlled manner. In their study, Cebolla et al. [18] found a correlation between internal WLOC and restrained eating behavior. In another study, it was observed that individuals with an internal locus of control tend to resist snacking until their cravings pass, consume less fat, reduce portion sizes, and decrease overall calorie intake [45]. Furthermore, it has been reported that the tendency to eat in response to negative emotions is associated with individuals’ WLOC [14]. All of these findings suggest that during menopause, anthropometric measurements may be influenced not only by biological mechanisms but also by psychological factors such as WLOC.

In our study, BF% and WHR were negatively associated with internal WLOC. Individuals with a high internal WLOC may be more likely to engage in healthy behaviors, such as regulating food intake and participating in physical activity [36]. This may support reductions in both BF% and WHR. Considering the adverse health effects of increased abdominal fat during menopause [46], the importance of internal WLOC is emphasized. One of the factor’s affecting BW is physical activity, and studies have reported that individuals aged ≥ 50 have insufficient physical activity. In fact, only a small proportion (7.2%) meet the recommendation of at least 150 min of moderate-intensity exercise per week, and activity levels tend to decline further after menopause [47, 48]. In our study, only 22.3% of the women reported engaging in regular exercise, indicating that women in menopause tend to be physically inactive. Therefore, promoting an internal WLOC that can support the prevention of physical inactivity is considered important to facilitate weight management in women during menopause.

Our logistic regression analysis results showed that a high chance WLOC is a significant risk factor for high BMI, meaning that women who believe their BW changes by chance are more likely to be overweight. Gruszka et al. [39] found that overweight and obese women with an external locus of control more frequently experienced a chance locus of control. Therefore, an external locus of control shaped by others is considered a potential risk factor for the development of obesity. In addition, providing quality health services to externally oriented individuals with a high chance locus of control may have positive effects on fostering an internal locus of control and supporting overall health [49]. The positive relationship between UE tendencies and high BMI indicates that UE is an independent risk factor for WG. The literature also shows that negative EE is common among middle-aged adults [50], and both UE and EE have been observed to be positively associated with BMI [42, 51]. These findings suggest that an external locus of control and loss of control overeating may be associated with WG during menopause.

During menopause, an increase in WHR is associated with greater abdominal fat accumulation, which is known to elevate obesity-related health risks [52]. Considering that menopause is a high-risk period for health, alternative approaches to prevent obesity in this group are important [53]. Regression analysis showed that an internal WLOC was protective against both high WHR and high BF%. These findings reveal that internal WLOC is associated not only with BMI but also with BF% and WHR, suggesting that it may be a key factor underlying weight management during menopause. If an individual believes they have control over their BW, can take active steps, and tends to maintain a healthy lifestyle through an internal locus of control, this may be effective in preventing abdominal obesity [54]. In a study conducted on middle-aged women, internal motivation was found to support sustainable changes in weight control behaviors [55]. In middle-aged women, motivational interviewing techniques focused on internal motivation have been found to produce significant reductions in BW, BF%, and waist circumference [56]. Individuals with an internal WLOC are more likely to evaluate information from their environment and take appropriate corrective actions [13]. Therefore, providing health education materials aimed at increasing internal WLOC in overweight or obese women may positively support both weight loss motivation and the changes in body composition that occur during menopause. Additionally, it is suggested that women with high BMI and BF% may benefit from having a balanced WLOC—being sufficiently internal to trust themselves in managing their weight, and sufficiently external to seek support from health professionals when needed—for maintaining well-being during menopause.

### Limitations

A limitation of our study is its cross-sectional design, which precludes causal inference. Participants were recruited from the health and social support centers of Alanya Municipality. Therefore, the sample consisted of individuals who were health-conscious, had access to healthcare services, and were socially active, while women who did not receive healthcare, were socially isolated, or had no access to such centers were not included in the sample. This limits the generalizability of our findings to all menopausal women. Future studies are recommended to use community-based sampling strategies. However, the limited number of studies assessing WLOC in our country, and the fact that our research is the first to examine the relationship between WLOC, eating behavior, and anthropometric measurements in menopausal women in Turkey, provide it with a unique value. This study may provide a new perspective for planning initiatives aimed at improving the health status of menopausal women.

## Conclusions

This study is among the pioneering research investigating the relationship between WLOC, eating behavior, and anthropometric measurements in menopausal women. The results of the study revealed that WLOC is associated with BMI, WHR and BF%. EE and UE behavior are high in menopausal women with a high BMI. Additionally, women with high BMI, WHR, and BF% have low internal locus of control. In short, it is important to assess the locus of control in managing BW in menopausal women. It is recommended that interventions for individuals with an internal locus be planned around self-managed nutrition and weight control, while interventions for individuals with an external locus should be personalized and implemented with support from health professionals.

## Data Availability

The data used and analysed during the study are available from the corresponding author upon reasonable request.

## Abbreviations

BF%: Body fat percentage
BMI: Body mass index
BW: Body weight
CR: Cognitive restraint
EE: Emotional eating
TFEQ-R21: Three-Factor Eating Questionnaire
T-MWLCS: Multidimensional Weight Locus of Control Scale
UE: Uncontrolled eating
WLOC: Weight locus of control
WG: Weight gain
WHR: Waist-to-hip ratio
